# Cadherins in early neural development

**DOI:** 10.1007/s00018-021-03815-9

**Published:** 2021-04-01

**Authors:** Karolina Punovuori, Mattias Malaguti, Sally Lowell

**Affiliations:** 1 Helsinki Institute of Life Science, Biomedicum Helsinki, University of Helsinki, 00290 Helsinki, Finland; 2 Stem Cells and Metabolism Research Program, Faculty of Medicine, University of Helsinki, 00290 Helsinki, Finland; 3 Centre for Regenerative Medicine, Institute for Stem Cell Research, School of Biological Sciences, University of Edinburgh, Little France Drive, Edinburgh, EH16 4UU UK

**Keywords:** Adhesion, Pluripotency, Neuroectoderm, Differentiation, Signalling

## Abstract

During early neural development, changes in signalling inform the expression of transcription factors that in turn instruct changes in cell identity. At the same time, switches in adhesion molecule expression result in cellular rearrangements that define the morphology of the emerging neural tube. It is becoming increasingly clear that these two processes influence each other; adhesion molecules do not simply operate downstream of or in parallel with changes in cell identity but rather actively feed into cell fate decisions. Why are differentiation and adhesion so tightly linked? It is now over 60 years since Conrad Waddington noted the remarkable "Constancy of the Wild Type” (Waddington in Nature 183: 1654–1655, 1959) yet we still do not fully understand the mechanisms that make development so reproducible. Conversely, we do not understand why directed differentiation of cells in a dish is sometimes unpredictable and difficult to control. It has long been suggested that cells make decisions as 'local cooperatives' rather than as individuals (Gurdon in Nature 336: 772–774, 1988; Lander in Cell 144: 955–969, 2011). Given that the cadherin family of adhesion molecules can simultaneously influence morphogenesis and signalling, it is tempting to speculate that they may help coordinate cell fate decisions between neighbouring cells in the embryo to ensure fidelity of patterning, and that the uncoupling of these processes in a culture dish might underlie some of the problems with controlling cell fate decisions ex-vivo. Here we review the expression and function of cadherins during early neural development and discuss how and why they might modulate signalling and differentiation as neural tissues are formed.

## A brief introduction to the cadherin superfamily

In the late 1970s, Masatoshi Takeichi proposed that cell–cell adhesion in Chinese hamster cells was mediated by two processes: one calcium-independent and the other calcium-dependent. The activity of the calcium-dependent process correlated with the presence of a 150 kDa molecule [4]. Several groups independently found that inhibition of the activity of this molecule with antisera and antibodies resulted in the inhibition of calcium-dependent adhesion, with evident changes in the morphology of antiserum/antibody-treated cells and embryos, and in the ability of these cells to reaggregate following disaggregation [5–13]. The identified glycoprotein was thus named “cadherin” (now E-cadherin, *Cdh1*) after the process of calcium-dependent adhesion it was found to mediate [12]. Similar experimental strategies were used to identify close family members N-cadherin (*Cdh2*) [14–16] and P-cadherin (*Cdh3*) [17].

**Fig. 1:**
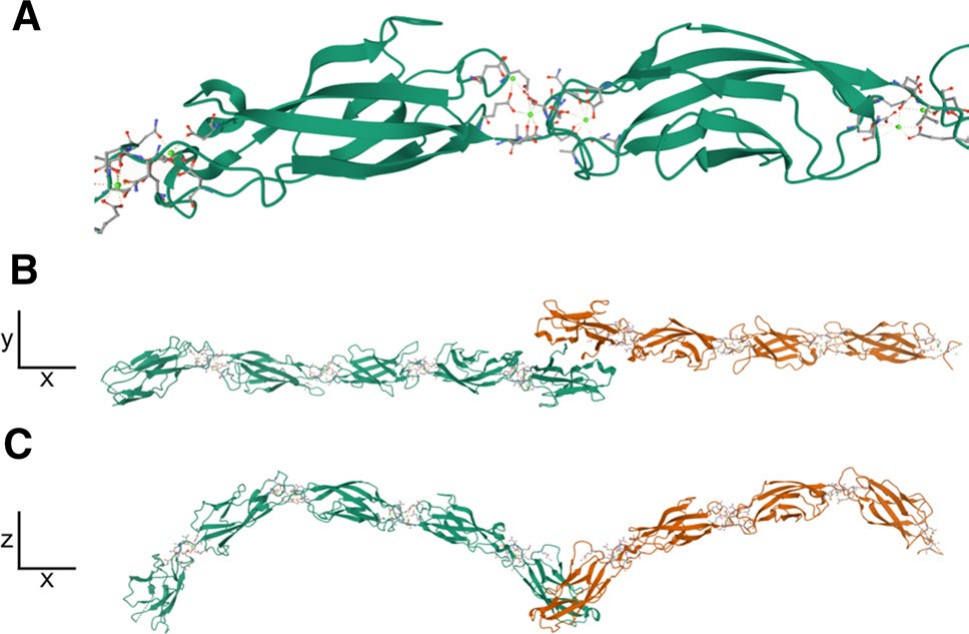
Structure of the cadherin domains of mouse Cdh1. **a** Two cadherin domains from mouse E-cadherin (EC2, EC3), forming seven-stranded β-sandwiches. Binding of three calcium ions (displayed in emerald green) is mediated at the aspartate and glutamate-rich interface of the two domains. Further calcium ions are visible at the interface of these domains with neighbouring cadherin domains. **b–c** *Trans*-interaction between two E-cadherin ectodomains (EC1-5 for green chain, EC1-4 for orange chain). A conserved residue (Trp2) in EC1 interacts with a hydrophobic pocket on the opposite EC1 domain. Crystal structure from [23] (PDB: 3Q2V) visualised with Mol*

The cadherin superfamily of proteins comprises more than 100 members in humans [18], with roles in several physiological processes including signalling, mechanotransduction, self-recognition and tumour suppression [19–22]. Proteins of this superfamily share an extracellular cadherin (CA) domain of approximately 110 amino acids in size (Interpro IPR039808). The CA domain is formed by seven β-sheets which arrange to form an Ig domain-like β-sandwich (Fig. 1a). It is found in tandem repeats and mediates both calcium binding and interaction with other cadherin molecules (Fig. 1b, c) [23].

**Fig. 2:**
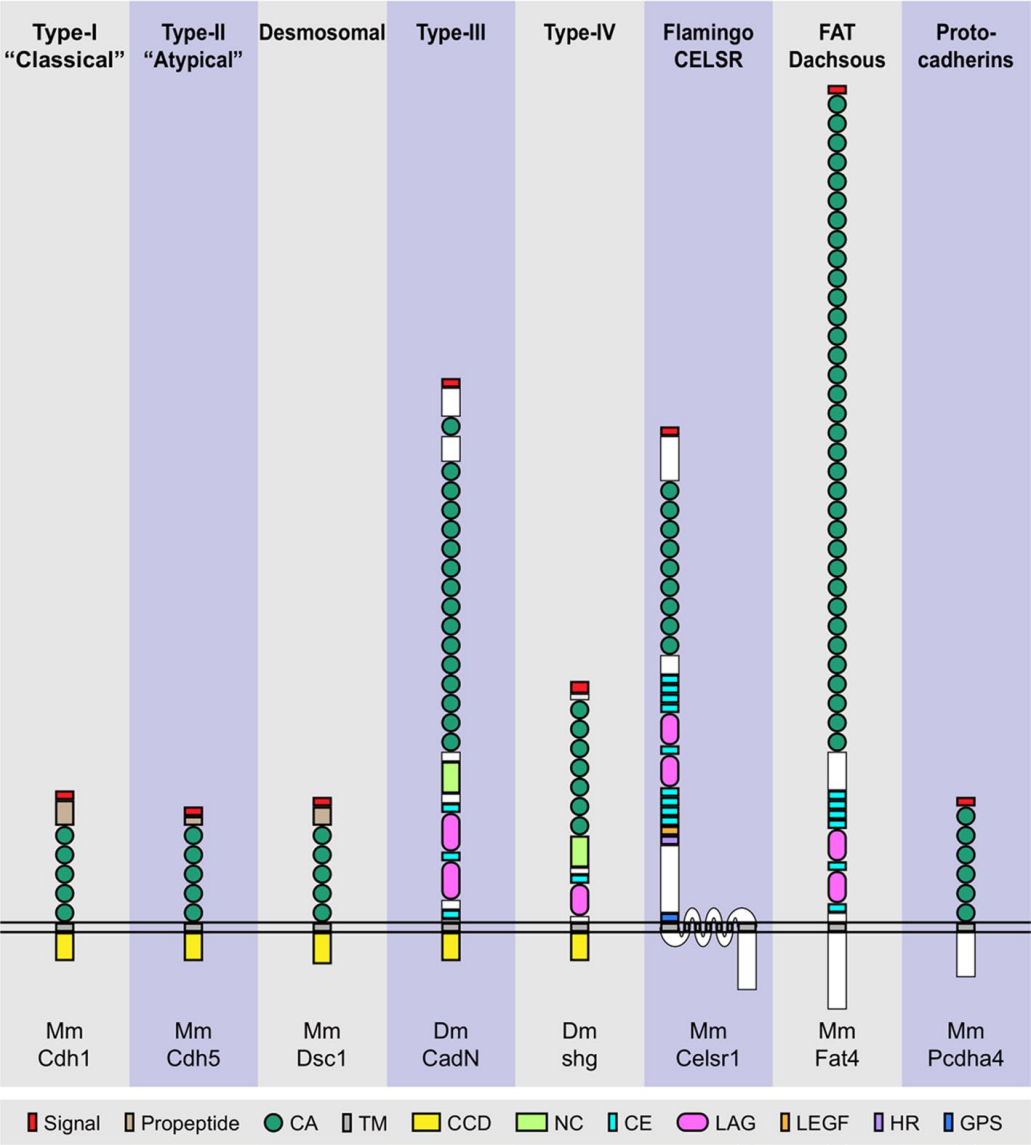
Cadherin subfamilies and representative members. Representative members of the eight cadherin subfamilies described in the text. The plasma membrane is illustrated as two parallel horizontal lines. *Mm* Mus musculus, *Dm* Drosophila melanogaster, *CA* cadherin domain, *TM* transmembrane domain, *CCD* cadherin cytoplasmic domain, *NC* non-chordate domain, *CE* cysteine-rich EGF-like domain, *LAG* laminin globular domain, *LEGF* laminin-type EGF-like domain, *HR* hormone receptor motif, *GPS* GPCR proteolytic site, white: other regions

The number of CA repeats and the composition of the remainder of the molecule vary greatly between different superfamily members and across species, and can be used to classify cadherin molecules into subfamilies. The following is a list of the main cadherin subfamilies and their properties, as comprehensively reviewed in [18, 20, 24–28] (Fig. 2).

Type-I or “classical” cadherins are located at adherens junctions, and are characterised by five extracellular CA (abbreviated to EC) domains, a transmembrane region, and an intracellular classical cadherin cytoplasmic domain (CCD, Interpro IPR000233), which binds armadillo family proteins β-catenin (*Ctnnb1*) and p120^ctn^ (*Ctnnd1*). The interaction with β-catenin links cadherins to α-catenin and the actin cytoskeleton, whereas p120^ctn^ is involved in cadherin turnover. *Trans*-interactions with other cadherins are mediated by conserved residues: a Trp in position 2 interacts with a hydrophobic pocket on the opposite N-terminal EC domain, which contains the conserved His-Ala-Val (HAV) motif [18, 26, 28, 29].

Type-II or “atypical” cadherins are structurally similar to type-I cadherins, but lack the HAV motif and have two Trp residues mediating *trans*-interactions (Trp2, Trp4) [24–26].

Desmosomal cadherins (desmogleins and desmocollins) are located at desmosomes. They have five EC domains, a transmembrane domain, and an intracellular CCD that interacts with armadillo family members plakoglobin and plakophilin, which link the cadherins to intermediate filaments via the protein desmoplakin [25, 26].

Type-III cadherins are not found in mammals other than the platypus. They have a variable number of EC domains followed by a conserved primitive classic cadherin domain (PCCD), composed of a “non-chordate” domain (NC), a cysteine-rich EGF-like (CE) and a LAG (laminin globular/LamG) domain. They have a transmembrane region and a CCD. Their EC1 domains lack conserved Trp residues but share a conserved Tyr in position 5 that may mediate *trans*-interactions [18, 24, 25].

Type-IV cadherins are found in arthropods and comprise seven EC domains followed by LAG and CE domains, a transmembrane region and a CCD [18, 25].

The Flamingo/CELSR (cadherin EGF LAG seven-pass G-type receptor) subfamily members have an extracellular region composed of nine EC domains followed by several LAG and CE domains, a laminin-type EGF-like domain, a hormone receptor domain and a GPS (GPCR proteolytic site) motif. This structure is reminiscent of the extracellular region of type-III cadherins, with which they also share the Tyr5 residue in EC1. They are highly unusual cadherins in that they have a 7-pass transmembrane (7TM) domain, which is why these cadherins are also referred to as the 7TM subfamily [18, 24, 25, 28].

The FAT, FAT-like and Dachsous group comprises proteins with large extracellular domains of up to 34 EC repeats, followed by LAG and CE domains for FAT and FAT-like cadherins, a transmembrane domain and a conserved C-terminal domain [18, 25].

Protocadherins are a large cadherin subfamily. They are subdivided into non-clustered protocadherins (NC-Pcdh) and clustered protocadherins (C-Pcdh). In human and mouse, C-Pcdh genes are transcribed from three adjacent gene clusters (Pcdh α, β and γ), with alternative splicing generating over 50 proteins with different N-termini and constant α, β and γ C-termini. The extracellular domains of both NC- and C-Pcdh contain 6/7 EC repeats with sequence similarity to the FAT/Dachsous group. They lack a conserved Trp2 residue, and are reported to interact in *trans* by means of anti-parallel interfacing of several EC domains. They share a transmembrane domain and conserved intracellular motifs. They are expressed in the mammalian brain, where they act as a cell-surface neuronal barcode to avoid self-synapsing [18, 20, 24, 27].

## An evolutionary perspective

Cadherins have classically been described as molecules that regulate calcium-dependent cell adhesion in metazoans [30–33]. However, cadherins containing EC domains, EGF-like domains and a single pass transmembrane domain have been identified in the genome of the unicellular filasterean *Capsaspora owczarzaki* and of several choanoflagellates, implying cadherins are a shared feature of Filozoa [34, 35]. A classical-like cadherin similar to Type-IV superfamily members, containing both EC repeats and a CCD was identified in the sponge *Oscarella carmela*, suggesting that interaction with armadillo family proteins evolved in an ancestral metazoan [35, 36].

**Fig. 3:**
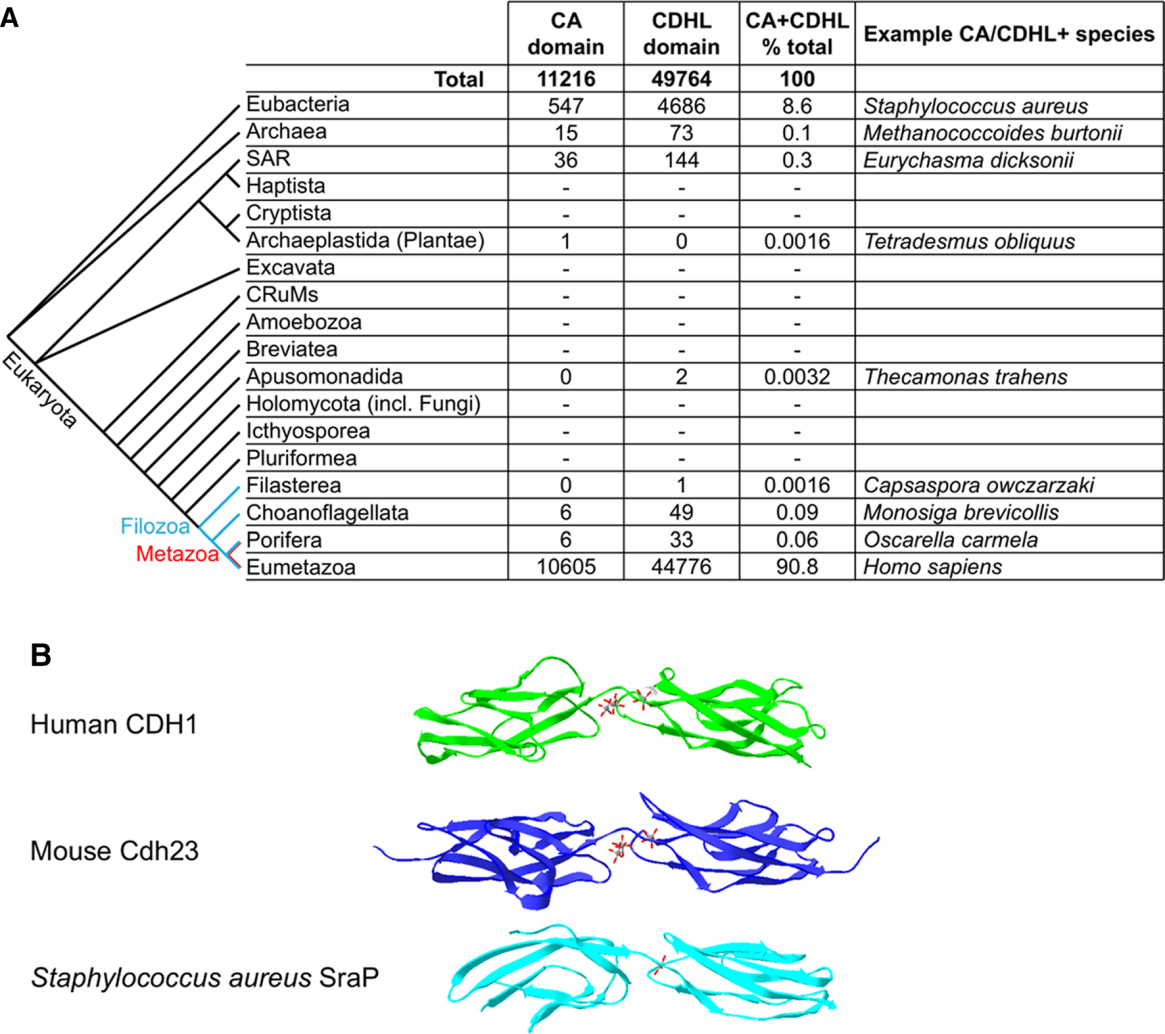
Phylogeny and structure of cadherin and cadherin-like domains. **a** Number of InterPro database entries for “Cadherin” and “Cadherin-like” protein domains, sorted taxonomically. Phylogenetic relationships of the described clades are based on [43–45]. InterPro data retrieved in May 2020. **b** Structural comparison of two CA domains of human CDH1 with two CDHL domains of mouse Cdh23 and Staphylococcus aureus SraP. Calcium ions are displayed as wireframe structures. Crystal structures from [46] (PDB: 3MVS), [47] (PDB: 2O72), [42] (PDB: 4M03), visualised with Swiss-PdbViewer [48] and aligned with the CA/CDHL domains on the right of the image in similar orientation

Pinpointing the evolutionary origin of cadherins, however, is not straightforward: proteins containing cadherin and cadherin-like domains have also been identified in bacteria and archaea [37–41]. The InterPro protein family database has over 60,000 combined entries for “Cadherin” (IPR039808, CA) and “Cadherin-like” (IPR002126, CDHL) domains. 90.8% of entries are specific to Eumetazoa, 8.6% to Eubacteria, 0.1% to Archaea, with the remainder spread amongst other eukaryotic clades (Fig. 3a).

The crystal structure of a *Staphylococcus aureus* protein containing CDHL domains [42] reveals that CA/CDHL domains have remained broadly structurally conserved throughout evolution, with calcium atoms bound at the interface of two Ig domain-like β-sandwiches (Fig. 3b). Variations in amino acid composition and in rotation angle between adjacent CA/CDHL domains may account for differences in the number of calcium ions bound at the interface of these domains (three for mammalian cadherins, one for *S. aureus* SraP).

Despite their presence in bacteria, archaea and Filozoa, CA/CDHL domain-containing proteins are not ubiquitous throughout eukaryotes. Aside from Filozoa, they are found almost exclusively in SAR, a large group of diverse species which diverged from Filozoa early in eukaryotic evolution (Fig. 3a). Two CDHL InterPro entries are recorded for the apusomonadid *Thecamonas trahens *[36], and one CA entry for plants (the green alga *Tetradesmus obliquus*). The *T. obliquus* entry is unreviewed, so it is unclear whether this protein truly contains CA domains.

This seemingly desultory phylogenetic distribution of CA/CDHL domain-containing proteins may be explained by horizontal gene transfer events. Ancient Filozoa were likely to be bacterivorous [49]. Similarly, *T. trahens* feeds on bacteria and on other flagellates [50]. These organisms could have therefore incorporated prokaryotic CA/CDHL-encoding DNA into their genomes. King and colleagues [35] suggest this is how bacterial cohesin domains may have been acquired in the choanoflagellate and Porifera coherin subfamily of cadherins.

The reverse process is also conceivable, with commensal, parasitic or pathogenic prokaryotic and SAR species potentially acquiring CA/CDHL domains from Filozoa. Proteins containing these domains could in turn support interaction with host cell surfaces, as suggested by Gachon and colleagues for the nonagonal family of SAR cadherins [51]. There are several examples of pathogenic and parasitic prokaryotic and SAR species with CA/CDHL domains (e.g., *Pythium insidiosum*, *S. aureus*, *Vibrio cholerae*) [39, 42, 51], but many prokaryotes and SAR species which are non-symbiotic and non-pathogenic to Filozoa also contain CA/CDHL domains (e.g., *Aureococcus anophagefferens*, *Methanococcoides burtonii*, *Rhodopirellula baltica*) [41, 51, 52].

Despite the uncertainty underlying the evolutionary origin of the cadherin domain, its ability to bind calcium appears to remain a defining feature throughout the tree of life [37, 40, 42, 53]. Aside from regulating adhesion in Metazoa and in some prokaryotes [33, 40], it has been suggested that non-metazoan proteins containing CA/CDHL domains may regulate cell–cell interactions in feeding processes, pathogen-host interaction and cell aggregation events. When considering the role of cadherins during neural development, we should bear in mind that they may have acquired multiple functions over evolutionary time and may be acting as more than just adhesion molecules.

## Early neural development

During early embryonic development, the fertilized egg undergoes several rounds of cell division, lineage segregation, symmetry breaking, and axis specification, generating the pluripotent epiblast and culminating in gastrulation and the formation of three germ layers that will go on to generate all embryonic tissues. These processes have been extensively reviewed elsewhere [54, 55], and here we will focus on subsequent neural development.

### Neurulation

**Fig. 4:**
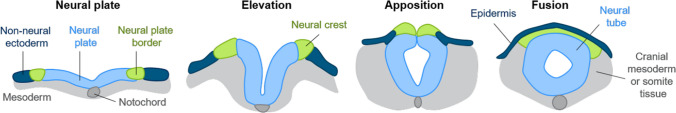
Generalised morphological events of neurulation leading to neural tube closure. Transverse view. Neurulation begins in the flat neural plate, which generates lineages of the central nervous system. It is flanked on either side by the neural plate border and non-neural ectoderm. As neurulation progresses, the neural plate thickens, stratifies and begins to fold, while a ventral hinge point is formed at the notochord, a mesodermal tissue. The neural plate borders elevate, becoming the neural crest. As the neural tube fuses dorsally, neural crest cells migrate out of it, going on to form lineages of the peripheral nervous system. Closure of the nascent neural tube disconnects it from the overlying epidermis

From gastrulation onwards, cells of the anterior epiblast that do not ingress through the primitive streak go on to form ectodermal tissues, giving rise to neurectoderm, which generates the nervous system, and surface ectoderm [56, 57]. Neurulation refers to the process of generating the neural tube, a tubular epithelial structure, out of the flat epithelial sheet of the neural ectoderm (Fig. 4). While anatomical differences exist between various species, modes of neurulation are generally conserved across amniotes, *Xenopus*, zebrafish and *Amphioxus* (reviewed in [58]).

Neurulation begins with the formation of the neural plate, which initially consists of a layer of neuroepithelial cells. As development progresses, these cells multiply, causing the neuroepithelium to thicken and stratify [59, 60]. The neural plate is flanked by a population of cells called the neural plate border, which distinguishes the neural plate from the rest of the ectoderm. The neural plate border will go on to form the neural crest, giving rise to the peripheral nervous system as well as several non-neural cell types including melanocytes, smooth muscle cells and, in the anterior, bone and cartilage [61]. Ectodermal tissues lying outside the neural plate and neural plate border will give rise to epidermis.

As the neural plate increases in size, it begins to fold, initiating the formation of the neural tube. Ventral to the neural plate lies the notochord, a mesoderm-derived embryonic tissue that serves as the precursor to the nucleus pulposus within the vertebral column. The neural plate forms a single ventral hingepoint directly above the notochord. Meanwhile, two dorsal hinge points are formed at the neural crest, forming a central ‘valley’ called the neural groove. The neural crest hinge points then converge towards each other, bringing the contralateral dorsal ends of the neural groove together, allowing them to fuse to form a closed tube. The closure of the neural tube detaches the neural ectoderm from the epidermis, which now dorsally overlies the neural crest and the nascent neural tube. Depending on the organism and the location along the anterior–posterior axis, the neural crest cells undergo an epithelial to mesenchymal transition (EMT) and migrate dorsally from either the converging neural folds or from the roof of the nascent neural tube [59, 60].

The potencies of the epiblast regions that give rise to neurectoderm have been investigated using explant culture studies. In gastrulating mouse embryos at E6.5, the anterior epiblast can give rise to all three germ layers [62, 63]. Within half a day however, cells in this region become restricted to ectoderm but retain potency to become either epidermal or neural lineages, determined by either the presence or absence of bone morphogenetic protein 4 (BMP4), respectively [64, 65]. At E7.5, the positional identity within the ectoderm defines cellular potency: the proximal region of the ectoderm is restricted to a surface ectodermal fate, while more distal regions of the anterior neurectoderm will give rise to the central nervous system [65–67].

Neuroepithelial precursor cells can be derived by directed differentiation from mouse embryonic stem cells (ESCs) [68], human ESCs [69, 70], and induced pluripotent stem cells (iPSCs) [71] by culture in basal medium with no exogenous growth factors [72]. Transcription factors that drive the formation of the anterior neuroectoderm include Sox2 [73, 74], Zfp521 [75], Pou3f1/Oct6 [76], Sip1[77], FoxD4 [78], and the homodimerised form of E2A [79].

### Signalling pathways controlling anterior neural specification

In vivo, neural fate specification depends on the inhibition of anti-neural signals BMP, Nodal, and WNT (reviewed in [80]). This has led researchers to propose a “neural default model”, which argues that pluripotent cells adopt a neuroectodermal fate unless specified otherwise. This is supported by the observations that early postimplantation embryos lacking Nodal [81] or BMP signalling [82] undergo premature and ectopic neural specification.

Pluripotent cells in culture, like their in vivo counterparts, progress to a neural identity when deprived of signalling inputs from the BMP [70, 83], Nodal [70], and Wnt [84, 85] pathways. Experiments using inhibitors of the fibroblast growth factor (FGF) pathway led to the proposal that autocrine FGF may be required for the acquisition of neural identity by mouse embryonic stem cells in culture [68], in keeping with findings in chick embryos [86, 87]. Later studies clarified that FGF can facilitate exit from naive pluripotency and subsequent induction and/or maintenance of formative [88] and primed [89–92] pluripotent states representative of post-implantation epiblast, but FGF must then be suppressed in order for primed pluripotent cells to efficiently acquire a neural identity [89, 93, 94]. Furthermore, embryos lacking BMP signalling default to a neural identity even when exposed to inhibitors of FGF signalling soon after implantation [82]. FGF signalling therefore appears to play a part in the exit from naive pluripotency and maintenance of the primed pluripotent state, but is then dispensable for neural specification. Additional factors, for example Notch signalling, can come into play to help coordinate the timing of neural fate specification between neighbouring cells possibly by dampening anti-neural signalling pathways [95], and unknown signals from adjacent tissues can refine the position of the neural plate boundary [96, 97].

In summary, the transition of primed pluripotent cells into anterior neuroectoderm is facilitated by inhibiting BMP, Nodal, Wnt, and FGF signalling pathways, while poorly understood local cell interactions signals seem to tune the response to these signals to refine the timing and position of neural fate specification.

### Neuro-mesodermal progenitors

In 2009, a single-cell clonal analysis of the mouse embryo revealed a distinct population of cells arising during early gastrulation that could give rise to both neural and mesodermal lineages [98]. These neuro-mesodermal progenitors (NMPs) are a transient population of cells that emerges in the mouse at head-fold stage (around E7.5) and persists until E13.5 [99, 100], long after other organ systems become specified. They largely drive the elongation of the anteroposterior axis during development and give rise to the neural tube and paraxial mesoderm [98].

In several vertebrates, including mouse, chick, and zebrafish, NMPs can be identified by the co-expression of the transcription factors T (Brachyury) and Sox2 [100–103]. Cells that subsequently differentiate into neural lineages downregulate T but maintain Sox2 expression [104–106], while those committing to mesoderm downregulate Sox2 and upregulate Msgn1 and Tbx6 [99, 107]. T and Sox2 play antagonistic roles in NMP lineage specification, with T being essential for NMP maintenance and promoting mesodermal fate, while Sox2 promotes neural fate acquisition. Tbx6 steers cells towards a paraxial mesoderm fate [108].

The induction, maintenance and differentiation of NMPs is governed by WNT and FGF signalling, and embryonic regions containing NMPs express ligands for both of these pathways [109–112]. In the absence of components of the WNT or FGF signalling cascades, the embryo becomes truncated and ectopic neural tissue forms in place of posterior paraxial mesoderm, demonstrating that both of these signalling inputs are required for mesoderm specification and axis elongation, but are dispensable for neural commitment [100, 109, 112–115]. For a more detailed review of signalling events during specification and differentiation of neuromesodermal progenitors, see [105].

## Cadherin expression patterns in neural development

Changes in cell identity are frequently accompanied by switches in expression of cadherins. For example, a switch from E-cadherin to N-cadherin is associated with the EMTs that accompany the formation of mesoderm or neural crest and with the dysregulation of tissue structure in tumorigenesis [116]. The emergence of the neural plate from the epiblast is not a classical EMT event but does bear a subset of EMT hallmarks, including loss of E-cadherin, gain of N-cadherin and upregulation of vimentin [117, 118]. Here we summarise changes in cadherin expression in the early stages of vertebrate development.

### E-cadherin and N-cadherin

E-cadherin is the predominant cadherin expressed in pluripotent cells immediately prior to neural specification. In mice, it is present in the unfertilized egg as both mRNA and protein [5, 119]. At early stages of development, there is additionally significant maternal contribution of the protein to facilitate blastomere adhesion and embryonic compaction [120, 121]. E-cadherin knockout results in embryonic lethality at the morula stage (E2.5 in mouse) due to defects in morula compaction and a failure to segregate the trophectoderm and inner cell mass [121–126]. In the chick, E-cadherin is widely expressed in the epiblast of the early conceptus [61, 127]. In Xenopus, the protein first becomes expressed in ectoderm following the blastula stage [128–130].

At gastrulation, the mesodermal cells ingressing through the primitive streak are the first to lose E-cadherin expression as they begin to undergo EMT [131–133]. Strong E-cadherin expression is maintained in the anterior neurectoderm and neural plate until the later stages of neurulation. Early studies of the avian embryo indicated a lack of E-cadherin in the neural tube at HH10 [127, 134], though more recent studies have shown that the protein remains expressed at this stage, albeit at lower levels than in the overlying ectoderm [61, 135].

As pluripotent cells transit to a neural fate, they also upregulate N-cadherin. In mice, N-cadherin can first be observed in the neural plate at E7.5 [136], and by E8.5, it is widely expressed in the neural tube [137]. In the avian embryo, the earliest expression of N-cadherin in the ectoderm is in the neural plate at HH7, and expression persists in the neural plate and the closed neural tube [16, 127, 135].

The expression patterns of E- an N-cadherin in the neural crest appear more dynamic. In the mouse embryo, as neural crest cells delaminate they undergo EMT, downregulating E-cadherin and upregulating N-cadherin in its place, to acquire a migratory phenotype [61, 135]. In the chick embryo, E-cadherin expression is maintained in the neural crest, while the downregulation of N-cadherin is required for the delamination of cells in this structure [61, 135]. However, this functional patterning appears species-dependent, as it differs from that observed in Xenopus [138] or zebrafish [139].

In *Xenopus*, ectoderm is specified into dorsal neural ectoderm at the end of gastrulation, and this is where N-cadherin is first expressed, concomitantly with the downregulation of E-cadherin. The non-neural ectoderm, fated for epidermis, retains E-cadherin expression [130].

E- to N-cadherin switching has also been observed during the maturation of neuromesodermal progenitors. During axial elongation in the mouse embryo, NMPs mature while maintaining bipotency to generate both neural and mesodermal daughter tissues in the posterior regions of the trunk. This maturation is accompanied by a switch from epithelial to mesenchymal gene expression similar to a partial EMT, including a switch from E- to N-cadherin expression [140].

### Other cadherin family members

In mice, P-cadherin (Cdh3) is first detected in the E4.0 trophectoderm but not in any embryonic tissues until later in development [141, 142]; it is not expressed in the neural ectoderm, but is instead found in non-neural ectoderm and other tissues [143]. In the chick, however, it has been suggested that P-cadherin is the predominantly expressed cadherin in the epiblast, reminiscent of E-cadherin expression in the early mouse embryo. This raises the possibility of an evolutionary exchange of E-cadherin for P-cadherin between mammals and birds, similar to the functional differences between the transcription factors Snail and Slug between the two species in EMT-like morphological events in early development [144, 145].

In the chick, K-cadherin (Cadherin-6B) is first expressed in the neural plate border at HH6, and subsequently plays a critical role in the specification and delamination of the neural crest [61]. In mice, K-cadherin (Cdh6) becomes detectable from E7.5 in neuroepithelial cells in the prospective hindbrain region, with expression in the forebrain by E8.0. At E8.5, K-cadherin is downregulated in the neural plate and neural tube, with expression persisting in the neural crest and in a band of the hindbrain at the level of rhombomere 6 (r6), and in the migrating neural crest cells emerging from r6; these neural crest cells go on to contribute to the peripheral nervous system. At E12.5, K-cadherin is found in the developing brain (expressed in a complementary pattern with R-cadherin) and in the spinal cord [146, 147]. Inoue et al. [146] suggest that K-cadherin facilitates cell sorting through differential adhesion of specific neural structures. Similarly, in zebrafish, combinatorial codes of cdh2, cdh11, and pcdh19 define different dorsoventral domains in the developing spinal cord and help to confer robust patterning by preventing the intermingling of cells from adjacent domains [148].

In mice, R-cadherin (Cdh4) is present in the neurectoderm of the developing midbrain at E8.5 [149]. Later, at E12.5, this cadherin is found in the lateral cortex of the brain in a complementary pattern with K-cadherin [146, 147]. In the chick, it is also detected in the neuroepithelium of the forebrain at E5 during later neural patterning [150].

Expression of cadherins 5 (VE-cadherin), 8, 9 (T1-cadherin), and 10 (T2-cadherin) has not been reported in the early mouse embryo [143], while cadherin 11 (OB-cadherin) is detectable in small amounts in the roof plate of the mouse neural tube [136]. Low levels of cadherin 7 transcript have been reported in the mouse neural tube at E8.5 [151]. In the chick, CDH4, 7, 8, 9, 11, 12 18 and 20 are expressed at later stages of neural patterning but not during early neurulation [152].

In Xenopus, C-cadherin (also called EP-cadherin) is the primary cadherin expressed from fertilized oocyte stage through to gastrula stages [130, 153, 154], and is critical for cell adhesion in the blastula [155]. C-cadherin continues to be expressed throughout neurulation in both neural and non-neural tissues [130]. U-cadherin is also expressed in all Xenopus cells during early development up to late neurula stage [156].

## Neural developmental phenotypes of cadherins

Cadherins play important roles at multiple stages of brain development [157, 158], spinal cord neurogenesis [159, 160], and neural crest formation [61] (reviewed in [161]). Classical cadherins regulate the migration of different neuronal subtypes to the correct cortical layers in the developing brain (reviewed in [162]) and help position motor neurons within the hindbrain [163] and ocular system [164]. In addition, cadherins are located at synapses [165] where they regulate both synaptic formation [166–168] and maturation [167]. Cadherins can facilitate contact inhibition of cell proliferation in some contexts [168] although it is not known whether this occurs during neural development. It is, however, clear that cadherins influence proliferation in the nervous system through other mechanisms, for example as a consequence of their ability to modulate the activity of various signalling pathways [169]. In this review, we focus on the roles of cadherins during early stages of neural development.

In mice, knockout of E-cadherin results in embryonic lethality due to a failure in compaction of the morula and formation of trophectoderm [124, 170]. This phenotype has been attributed to the loss of adhesion and signalling functions of E-cadherin during the earliest stages of embryogenesis. Epiblast-specific replacement of E-cadherin with N-cadherin in mice leads to embryonic death at E8.5 due to improper growth and degeneration of the epiblast. Gastrulation is initiated in these embryos and all germ layers maintain their differentiation capacity, but mesoderm formation is compromised due to impaired BMP signalling [171]. These findings again implicate a specific role for E-cadherin in modulating signalling during embryonic development that may be independent of adhesion.

N-cadherin knockout is embryonic lethal in mice at around E10 due to heart defects; embryos also have malformed somites and a “wavy” neural tube, defects which appear to be caused by impaired cell–cell adhesion [137]. Work in ascidian embryos suggests that N-cadherin facilitates the directed forces that drive neural tube closure [172]. However, N-cadherin-null cells can form neural rosettes and adopt a neural tube-like organisation in teratoma assays, suggesting that the protein is not essential for the formation of simple neural structures. Cells lacking both N-cadherin and P-cadherin can give rise to rosettes but not neural tube-like structures, suggesting a certain level of functional redundancy between these two cadherins in the mouse, since embryos with knockout of P-cadherin alone are viable and have normal neural organisation [173, 174].

In Xenopus, depletion of N-cadherin in the neural plate results in abnormal invagination of the plate during neurulation (similar to spina bifida), caused by failure in actin assembly resulting in insufficient forces to direct folding of the neural plate [130]. While some bending in the neural plate can be observed in these embryos, the force for this bending is supplied exclusively by pushing forces from the non-neural ectoderm. Depletion of E-cadherin in the same structure in Xenopus embryos causes impaired spreading of the non-neural ectoderm and results in failure of neural fold closure, likely due to defects in E-cadherin-dependent cortical actin assembly and resulting in impaired movement of epidermal cells [130]. Neither of these phenotypes can be rescued by overexpressing the other cadherin, highlighting cadherin-specific roles in mechanical function during neurulation. In zebrafish, deletion of N-cadherin gives rise to a characteristic T-shaped neural tube due to a defect in convergent cell movements during neurulation [175, 176].

Overexpression of E-cadherin or N-cadherin in xenopus embryos results in failure to form the neural plate, with embryos instead developing large cysts. At later stages, these embryos do not develop a visible anteroposterior or dorsal–ventral axis. The same phenotype is seen upon depletion of β-catenin, and appears to involve a failure of neural and dorsal mesodermal differentiation [155].

In cultured neural stem cells (NSCs), overexpression of E-cadherin causes a downregulation of N-cadherin, and conversely, RNAi-mediated knockdown of E-cadherin causes N-cadherin to be upregulated, suggesting that these two cadherins may repress each other’s expression. The same study found that overexpression of E-cadherin inhibits the migration of NSCs; effects on differentiation were not examined [177].

It is clear that cadherins are crucial for early neural development to proceed correctly, raising the questions of whether this requirement for cadherins is explained predominantly by their ability to modulate cell–cell adhesion to drive morphological changes [60], or whether they may also perform other functions that directly influence the differentiation process.

## Cadherins modulate neural differentiation

There is plentiful evidence that cadherins can protect or promote pluripotency in a number of contexts [178–185]. Here we will review the evidence that cadherins are also able to regulate the transition from pluripotency to neural identity.

Downregulation of E-cadherin is tightly correlated with a transition from pluripotency to neural identity in culture [186]. This timely loss of E-cadherin seems to be a limiting factor for cells to enter the neural lineage: experimental suppression of this adhesion molecule results in faster and more uniform neural differentiation [186].

What regulates the changes in cadherin expression that enable efficient differentiation? As discussed above, inhibition of BMP signalling is a key event that triggers neural differentiation [79, 82, 83, 87]. BMP also maintains high levels of E-cadherin in pluripotent cells [186]. The ability of BMP to maintain high E-cadherin seems to explain its ability to block neural differentiation; experimental suppression of E-cadherin is sufficient to overcome the inhibitory effects of BMP on neural differentiation if no other anti-neural signals are present, and if BMP levels remain below a particular threshold [186]. So, in situations where BMP levels are not too high, E-cadherin is the primary mediator of its anti-neural effects.

Higher doses of BMP do, however, have the additional ability to impose a posterior identity that favours mesodermal rather than neural priming of pluripotent cells. This second anti-neural effect of BMP seems to be independent of E-cadherin. This might explain why downregulation of E-cadherin does not result in neural differentiation in the posterior of the embryo, where several BMP family members (together with other anti-neural signals) are expressed at high levels [54].

It seems likely that gain of N-cadherin also influences the acquisition or stability of neural identity. Forcing premature N-cadherin expression can enhance neural differentiation in culture even in situations where E-cadherin initially remains present [187]. Manipulations of N-cadherin have also been used to demonstrate that cadherins can influence differentiation independently of any changes in adhesion. For example, N-cadherin can fully rescue the adhesion phenotype of E-cadherin mutant pre-implantation embryos, but does not rescue all defects in signalling and differentiation phenotypes [122, 187]. Similarly, premature activation of N-cadherin can result in pro-neural phenotypes in culture without any apparent effect on the spatial organisation or cohesion of cells [187].

These findings confirm that both E- and N-cadherin may contribute to the efficiency of neural differentiation. What might be the mechanisms by which cadherins influence cell fate? Developmental transitions are sensitive to changes in the forces that cells impose on their neighbours [188, 189] and by movements of cell populations relative to each other [190]. Both of these processes could be modulated by changes in cadherin expression. The work discussed above [122, 187] does, however, hint that at least part of the mechanism by which cadherins control differentiation may not be explained only by changes in the cell–cell adhesion. This might instead be explained by the ability of cadherins to directly interact with components of signalling pathways.

## Cadherins modulate signalling

Cadherins do more than just stick cells together, something that should perhaps not come as a surprise given that cadherins first appeared in unicellular organisms. As multicellular organisms evolved, it seems that cadherins may have acquired a diverse range of functions. For example, it is now well established that cadherins can bind to various components of signal transduction pathways to modulate signal responsiveness. This could provide an opportunity for cells to coordinate changes in morphology (influenced by changes in adhesion) with changes in cell identity (influenced by changes in signalling pathway activity). There have been a number of excellent reviews [161, 191, 192] covering ways in which cadherins can modulate signalling pathways. Here we outline a few examples that may be of particular relevance to early neural development.

Perhaps the most obvious candidate mechanistic link between cadherins and neural differentiation is the Wnt signalling pathway. Wnt signalling has multiple stage-specific effects on development of the nervous system, but its earliest effects seem to be anti-neural; Wnt activity needs to be suppressed if either pluripotent or neuromesodermal progenitor cells are to transit to a neural identity [84, 85, 100, 109, 112]. β-catenin is a component of adherens junctions and also a component of the Wnt signal transduction pathway, and so provides one possible mechanistic link between cadherins and Wnt signalling. In at least some contexts [155, 193] cadherins can titrate the amount of β-catenin that is available for mediating Wnt signalling, effectively acting as a ‘dampener' of the Wnt response. Moreover, it has been suggested that E-cadherin does not merely dampen Wnt activity but can also prime it for future activation [194], for example during mesoderm formation from pluripotent human cells [195]. A positive influence of E-cadherin on Wnt responsiveness is in keeping with the observation that loss of E-cadherin can decrease rather than increase Wnt responsiveness in a mammary epithelial cell line [196].

These findings raise the possibility that the switch from E- to N-cadherin may result in a switch in Wnt responsiveness. In support of this idea, E-cadherin and N-cadherin differ in their ability to influence Wnt activity during mesoderm formation in drosophila: E-cadherin, but not N-cadherin, can effectively suppress Wnt activity. Importantly, particular point mutations in E-cadherin can abolish effects on Wnt activity without affecting cell adhesion [197]. It therefore seems likely that the switch from E- to N-cadherin during neural differentiation might modulate receptiveness to Wnt independently from effects on cell adhesion.

When pluripotent cells are forced to experience a premature cadherin switch by replacing the coding sequence for E-cadherin with that for N-cadherin in mouse ES cells [123, 171, 187], this results in a strong reduction in β-catenin levels accompanied by an increase in the efficiency of neural differentiation. Furthermore, this forced cadherin switch enables cells to resist the anti-neural effects of exogenous Wnt. Surprisingly, however, this apparent “Wnt resistance” seems to operate via an indirect mechanism, because the transcriptional response to Wnt remained intact in cells that had undergone an enforced cadherin switch. Therefore, during neural differentiation of pluripotent cells in culture, the ability of cadherins to influence neural differentiation does not seem to be explained by their ability to modulate transcriptional activity via Wnt signalling. What other signalling pathways might be regulated by cadherins during early neural development? One promising candidate is the FGF signalling pathway.

There are a number of ways in which cadherins modulate FGF activity. FGF receptors (FGFRs) interact with N-cadherin, for example during neurite outgrowth, and may even mediate ligand-independent activation [52]. Similarly, N-cadherin can bind FGF receptors and potentiate FGF activity in breast cancer cell lines [198]. Perhaps most strikingly, mice engineered to express N-cadherin in place of E-cadherin in mammary epithelia exhibit constitutive FGF activity and pre-malignant growth [199].

**Fig. 5:**
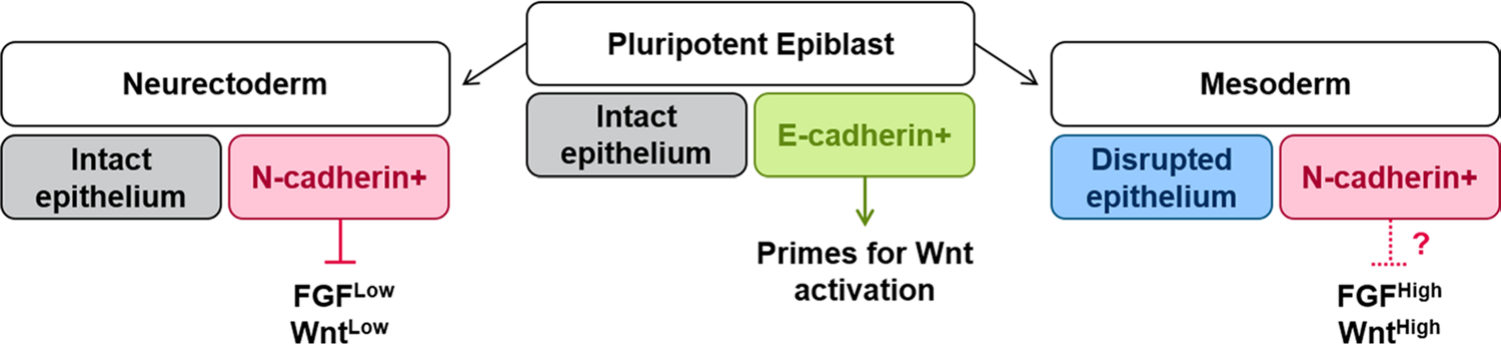
E-cadherin is exchanged for N-cadherin as the pluripotent epiblast forms neuroectoderm or mesoderm. E-cadherin in the epiblast may prime cells for future activation of Wnt [189, 197]. N-cadherin in the neuroectoderm dampens low levels of FGF that would otherwise have anti-neural activity [189]. It remains an open question whether the high levels of FGF or other differences in context in the emerging mesoderm override the FGF-dampening effects of N-cadherin

FGF can sustain primed pluripotency [90, 91] and suppress neural differentiation of epiblast cells [89, 94], at least in culture. N-cadherin has been reported to sustain FGF responsiveness to maintain pluripotency in EpiSCs [185], although the in vivo relevance of this is unclear given that N-cadherin is not readily detectable in the epiblast [187]. N-cadherin is, however, readily detectable at the onset of neural differentiation in culture and, as discussed above, is able to enhance the efficiency of the differentiation process. By measuring the activity of a panel of signalling pathways, Punovuori et al. [187] found that N-cadherin dampens the activity of pathways downstream of FGF receptors during early neural differentiation. Restoring FGF activity can reverse the pro-neural effects of N-cadherin. It seems therefore that N-cadherin has context-specific effects on FGF signalling, and in the context of neural differentiation it acts to dampen FGF activity and consequently enhances the transition from pluripotency to a stable neural identity (Fig. 5).

Much remains to be explored about the links between signalling pathways and neural development. For example, E-cadherin binds insulin-like growth factor 1 (IGF1) receptor and this interaction is critical for survival of cells in the preimplantation embryo [122]. IGF is also important for survival of neural progenitor cells, so it would be interesting to explore whether cadherins also influence IGF activity in the nervous system. In endothelial cells, VE-cadherin can bind and stabilise BMP receptors [200], making it tempting to speculate that other cadherins expressed before and during neural specification may also influence BMP signalling.

In summary, it seems clear that effects of cadherins on signalling are diverse and context specific. In the context of neural differentiation one might speculate that cadherins act as ‘dampeners’ of multiple signalling pathways and so help shield cells from anti-neural influences. Furthermore, a loosening of cell–cell contacts caused by changes in cadherin expression may interfere with juxtacrine signalling from surface-bound ligands, for example through the Notch receptor. It would be interesting to explore these ideas in future work.

## Perspectives

Why might it be useful for the cells of the embryo to use cadherins to help inform their differentiation decisions during early neural development? Neural tissue forms within an embryo that is growing and changing shape rapidly. Signalling molecules appear and disappear from different regions of the embryo over a short space of time as they are used and then re-used for successive differentiation decisions. In this constantly changing environment, it might be difficult to ensure that there are never any ectopic residual or premature signals that might disrupt neural specification.

Cadherin switching can dampen signal responsiveness. We propose that the embryo exploits this property of cadherins in a ‘belt and braces’ approach to guard against any ectopic signals that might otherwise mislead cells into the ‘wrong’ fate. One important open question is whether cadherins influence differentiation only cell-autonomously or whether they can also share this information with neighbouring cells, and so help coordinate neural differentiation across a local neighbourhood, perhaps propagating information from cell to cell as has been described during mesoderm formation [201].

Given that cadherins first appeared in unicellular organisms, they will have had ample time to acquire multiple functions, and therefore would be well placed to coordinate multiple events during the development of multicellular organisms. In this review, we have focused on the ability of cadherins to coordinate changes in adhesion with changes in signal response, but it remains possible that cadherins influence and coordinate other biological processes. It would be interesting to explore these ideas and test their relevance to neural development.

Can our understanding of cadherins in early neural development help us to gain better control over differentiation of cells in culture? For reasons still unknown, the timing of cadherin switching relative to neural specification becomes partially dysregulated in cultured cells; in the embryo, N-cadherin only becomes readily detectable in cells that have already switched on early neural marker Sox1, whereas in culture N-cadherin can be found in cells that retain pluripotency markers and lack neural markers [187]. Does this variability in cadherins explain variability in the differentiation response? There is some evidence that this is indeed the case: forcing a more uniform cadherin switch does seem to impose a more homogenous differentiation response, at least in monolayer culture [186, 187]. It would be interesting to explore whether manipulation of cadherins can also give better control over differentiation of cells in 3D or organoid culture in which cells have a more in vivo-like spatial organisation.

Our understanding of differentiation is gradually shifting from a view dominated primarily by signalling pathways, transcription factors, and chromatin structure to a broader understanding incorporating information from adhesion and morphology. As John Gurdon wrote, “most aspects of animal development seem to proceed by the cooperation of several contributory processes, no one of which is individually indispensable" [2]. Cadherins are unlikely to be essential instructors of neural differentiation decisions, but do seem likely to improve coordination of cell decisions across fields of cells, thus contributing to the fidelity of patterning in the early embryo. Cells, it seems, do better when they stick together.
